# Moderate traumatic brain injury triggers long-term risks for the development of peripheral pain sensitivity and depressive-like behavior in mice

**DOI:** 10.3389/fneur.2022.985895

**Published:** 2022-09-20

**Authors:** Gundega Stelfa, Baiba Svalbe, Edijs Vavers, Ilmars Duritis, Maija Dambrova, Liga Zvejniece

**Affiliations:** ^1^Laboratory of Pharmaceutical Pharmacology, Latvian Institute of Organic Synthesis, Riga, Latvia; ^2^Faculty of Veterinary Medicine, Latvia University of Life Sciences and Technologies, Jelgava, Latvia; ^3^Department of Pharmaceutical Chemistry, Faculty of Pharmacy, Riga Stradiņš University, Riga, Latvia

**Keywords:** long-term behavioral outcome, lateral fluid percussion injury, pain, depression, traumatic brain injury

## Abstract

As traumatic brain injury (TBI) is one of the major causes of permanent disability, there is increasing interest in the long-term outcome of TBI. While motor deficits, cognitive impairment and longer-term risks of neurodegenerative disease are well-established consequences in animal models of TBI, pain is discussed less often despite its high prevalence. The current study addresses the need to characterize the extent of chronic pain and long-term behavioral impairments induced by moderate lateral fluid percussion injury (latFPI) in mice up to 12 months post-TBI and evaluates the validity of the model. Adult male BALB/c mice were subjected to latFPI, and the results were compared with outcomes in sham-operated mice. Mouse behavior was assessed at 1 and 7 days and 1, 3, 6, 9, and 12 months post-injury using sensory-motor (neurological severity score, NSS), cold (acetone) and mechanical sensitivity (von Frey), depressive-like behavior (tail suspension), locomotor (open field), motor coordination (rotarod) and cognitive (Morris water maze, y-maze, passive avoidance) tests. Animals with TBI demonstrated significantly higher NSS than the sham-operated group for up to 9 months after the injury. Cold sensitization was significantly increased in the contralateral hind paw in the TBI group compared to that of the sham group at 3, 6, and 9 months after TBI. In the von Frey test, the withdrawal threshold of the contralateral and ipsilateral hind paws was reduced at 6 months after TBI and lasted for up to 12 months post-injury. latFPI induced progressive depressive-like behavior starting at 6 months post-injury. No significant deficits were observed in memory, motor coordination or locomotion over the 12-month assessment period. The present study demonstrates that moderate TBI in mice elicits long-lasting impairment of sensory-motor function, results in progressive depression and potentiates peripheral pain. Hence, the latFPI model provides a relevant preclinical setting for the study of the link between brain injury and chronic sequelae such as depression and peripheral pain.

## Introduction

Traumatic brain injury (TBI) is one of the leading causes of trauma-related permanent disability worldwide, especially in people under the age of 45 (1–3). Worldwide estimates suggest that more than 50 million new TBIs occur each year, with approximately 70–90% of patients having mild to moderate TBI (4). While many symptoms of mild to moderate TBI dissipate rapidly after injury, these patients frequently experience unusually high rates of chronic pain (5). It is widely recognized that the experience of pain is a frequent occurrence in TBI patients (6). In most of these instances, chronic pain is located in the head and is associated with brain tissue damage (7). Pain in TBI patients has also been reported in seemingly intact body regions, such as the upper and lower limbs (7, 8). However, chronic post-TBI pain in non-head body regions could also be associated with local injury (e.g., fractures, wounds), peripheral neuropathy or a related spinal injury (7). Therefore, the anatomical source of pain in TBI patients is often not identifiable when pain is chronic (9). To date, the mechanisms for chronic pain in TBI patients are largely unknown (7). Psychological disorders, including depression, following TBI are commonly reported comorbidities of posttraumatic pain (5). Depression and pain following TBI are believed to exacerbate each other, and both are rooted in common biological mechanisms, such us shared neurotransmitter pathways in periaqueductal gray, which is a key anatomic structure in the pain modulation system (10, 11). In animal models of TBI, the response to the peripheral pain reflex, such as mechanical and thermal sensitivity, has been studied up to 2 months after controlled cortical injury (CCI) and fluid percussion injury (FPI) (12). Only a small subset of experimental TBI models evaluated long-term consequences after injury (>6 months after TBI, see Supplementary Table S1). It has been demonstrated that mice subjected to cortical impact injury (CCI), which is often characterized by extensive brain tissue loss, demonstrate persisting behavioral deficits, such as depressive-like behavior, cognitive decline and motor dysfunction, up to 12 months after injury (13–16) (see Supplementary Table S1). However, more moderate CCI produces less extensive morphological damage (tissue loss at the cortical region) and less prominent behavioral deficits (17) (see Supplementary Table S1). Only a few studies have evaluated long-term behavioral changes in rats following FPI, mostly limited to single time points or memory evaluation (18–20).

We recently reported that CD-1 background mice develop neurological and cognitive impairments over a 12-month assessment period after lateral FPI (latFPI) (21). It should be noted that behavioral outcomes after TBI could depend on the background strains of mice (22). Most long-term follow-up TBI studies have been performed on C57Bl/6 background mice. It is known that different mouse strains vary in their inherent behavioral characteristics (23). For example, C57BL/6 and BALB/c mice exhibit differences in anxiety-like and depressive-like behavior, pain sensitivity, motor performance, learning and memory (24–27). The aim of the present study was to characterize the development of pain sensitivity and depressive-like behavior up to 1 year after the latFPI model in BALB/c background male mice. In addition, cognitive function, general activity and sensorimotor and coordination abilities were evaluated to provide a complete and relevant description of the long-term behavioral changes induced by TBI in mice. We found increased pain sensitivity with ongoing depressive-like behavior up to 1 year after latFPI in mice. The assessment and interplay between both conditions post-TBI should be more detailly investigated in the future preclinical and clinical studies, that may lead to the rational design of therapies that both reduce and improve functional outcomes after TBI.

## Materials and methods

### Animals

Twenty male Balb/c mice (Envigo, Venray, Netherlands) 10-week-old, weighing 20–26 g, were used in this study. All animals were housed under standard conditions (21–23°C, 12 h light-dark cycle) with unlimited access to standard food (Lactamin AB, Mjölby, Sweden) and water in an individually ventilated cage housing system (Allentown Inc., Allentown, New Jersey, USA). Each cage contained bedding of EcoPure™ Shavings wood chips (Datesand, Cheshire, UK), nesting material and a wooden block from TAPVEI (TAPVEI, Paekna, Estonia). For the enrichment, transparent tinted (red) non-toxic durable polycarbonate safe harbor mouse retreat (Animalab, Poznan, Poland) was used. The mice were housed with up to 5 mice per standard cage (38 × 19 × 13 cm). All studies involving animals were reported in accordance with the ARRIVE guidelines (28). The experimental procedures were performed in accordance with the guidelines reported in the EU Directive 2010/63/EU and in accordance with local laws and policies; all procedures were approved by the Latvian Animal Protection Ethical Committee of Food and Veterinary Service in Riga, Latvia.

### The lateral fluid percussion injury model

The lateral fluid percussion injury model was performed as previously described (29). Briefly, mice were anesthetized using 5% isoflurane dissolved in 100% oxygen. After the onset of anesthesia, the concentration of isoflurane was decreased to 2.5%. Before trauma induction, the mice received a subcutaneous administration of tramadol at a dose of 10 mg/kg (KRKA, Novo Mesto, Slovenia). A craniectomy was performed using a 3 mm (outside diameter) circular trephine over the parietal region, 2 mm posterior to bregma and 2 mm right of midline. The bone flap was gently pulled from the underlying dura, leaving it intact. An injury hub (Leur-Loc syringe hub cut from a 23-gauge needle) was affixed to the skull using dental cement and then filled with sterile saline. An injury of 0.83 ± 0.09 ATM was induced using a fluid percussion device connected to a pressure measurement instrument (Model FP 302, AmScience Instruments, Richmond, VA, USA). Duration of apnea was monitored immediately after the injury. After the injury, the bone flap was put back, the wound was closed, and the mouse was placed in an awakening cage. Sham-injured animals underwent the same procedures as the animals in the latFPI group, except for trauma induction.

### Behavioral tests

**Figure 1:**
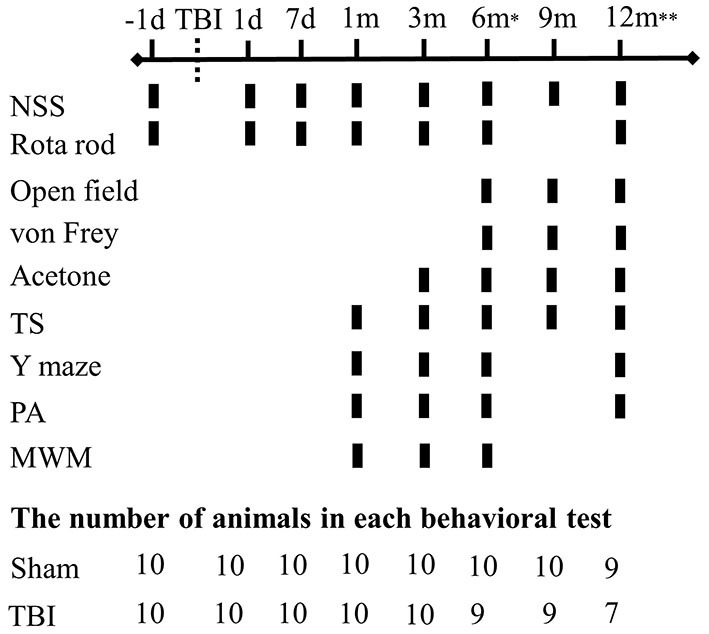
Experimental schedule for behavioral evaluation. Functional deficits were assessed at multiple intervals up to 12 months post-injury (*n* = 10/group). (d-days, m-months, NSS-neurological severity score, TS-tail suspension, PA-passive avoidance, MWM-Morris water maze). ^*^One animal died in the TBI group. ^**^Two animals died in the TBI group and one animal died in the sham group.

Animals were randomly separated into two experimental groups, sham-operated (*n* = 10) and latFPI (*n* = 10). The body weight of the mice was measured before injury (baseline measurement), on the first 3 days after the injury and then weekly throughout the study as a measure of general health. Behavioral tests were performed at baseline, 1 and 7 days, and 1, 3, 6, 9, and 12 months after the injury. Behavioral testing was conducted by an experimenter blinded to the study group. Tests were performed from the least aversive to the most aversive (neurological severity score < rotarod < open-field < Y-maze < acetone < von Frey < passive avoidance < tail suspension < Morris water maze test) with at least 2-day intervals among them to relieve animal's stress. The timeline of experimental procedures and the number of animals at each behavioral testing time point are given in Figure 1.

#### Neurological severity score (NSS)

The neurobehavioral status of mice was assessed using the NSS as previously described (29). The NSS consisted of nine individual clinical parameters, including tasks on motor function, alertness and physiological behavior. The mice were assessed for the following items: the presence of paresis; impairment of seeking behavior; absence of perceptible startle reflex; inability to get down a rectangle platform (34 × 27 cm); inability to walk on 3-, 2-, and 1-cm wide beams; and inability to balance on a vertical beam of 7 mm width and a horizontal round stick of 5 mm diameter for at least 15 s. If a mouse showed impairment on one of these items, a value of 1 was added to its NSS score. Higher scores on the NSS thus indicate more significant neurological impairment.

#### Electronic von frey test

Mechanically evoked pain-like behavior (mechanical sensitivity) was measured using an electronic von Frey plantar aesthesiometer (Dynamic Plantar Aesthesiometer, Model 37400-002, Ugo Basile, Gemonio VA, Italy) as previously described (30). During the test, the mice were placed on a metallic grid floor in an individual plastic observation chamber (10 cm W x 10 cm L x 14 cm H) and allowed to habituate to the environment for 30 min. The von Frey filament was applied to the midplantar surface of the hind paw. The withdrawal threshold was defined as the average latency time (s) required for causing withdrawal of the stimulated paw over three trials.

#### Acetone evaporation test

Cold-evoked pain-like behavior (cold sensitivity) was measured by applying a drop of acetone to the plantar surface of the hind paw (31). 1 day before the experiment and on the experimental day, mice were placed in individual plastic cages (10 cm W × 10 cm L × 14 cm H) on an elevated wire mesh metal floor and habituated for at least 30 min. On the experimental day, acetone was loaded into a 1-ml syringe without a needle. Air bubbles were cleared from the syringe before acetone application. One drop of acetone (approximately 40 μl) was applied to the paw from the bottom of the elevated cage through the wire mesh metal floor and onto the plantar surface of the hind paw. For 45 s, the mouse was scored on lifting, biting, and licking the paw. Each hind paw was measured twice with an interstimulation interval of approximately 15 min. The reaction duration was measured and analyzed as a cumulative reaction time.

#### Rotarod test

A rotarod test with slight modification was used to measure motor coordination (32). Briefly, mice were pre-trained (5 rpm) on the rotarod apparatus (Model 47650, Ugo Basile, Gemonio VA, Italy) with two sessions per animal, each lasting 240 s. On an experimental day, mice were placed on the rod with an accelerating rotating speed from 5 to 25 rpm for 240 s with a 30 min rest between trials. Time spent walking on the accelerating rotarod before falling off was measured. The mean of two trials was calculated for each mouse.

#### Passive avoidance test

The passive avoidance test was performed as previously described (32). Briefly, on the training day, each mouse was individually placed in the light compartment of an apparatus with no access to the dark compartment and allowed to explore for 60 s (Ugo Basile, Comerio, Italy). After this time, the sliding door (4 × 4 cm) was automatically opened, and the mouse was allowed to cross over into the dark compartment. Upon entering the dark compartment, the door was closed, and the mouse received a shock of 0.3 mA for 3 s. After 20 s, the mouse was returned to its home cage. A retention test was performed on the next day (24 h later) without any shock. The time to enter the dark compartment was recorded as the retention latency. The maximum retention latency was set at 540 s.

#### Y-maze test

Working memory performance was assessed by recording spontaneous alternation behavior in a Y-maze, as previously described (33). The experiment was conducted in a dimly red-lit room. The mice were individually placed at the end of one arm in a symmetrical Y-shaped runway (arm's length 35, width 5, height 21 cm) and allowed to explore the maze for 5 min. The total number and sequence of arm entries were manually recorded, and the percentage of alternation behavior was calculated.

#### Morris water maze test

The Morris water maze (MWM) was used to assess spatial cognition as previously described (34). The MWM apparatus was a blue-painted circular fiberglass pool (height: 60 cm, diameter: 150 cm) located in a test room surrounded by several cues. The experiment was conducted in a dimly red-lit room. The pool was virtually divided into four equal imaginary quadrants identified as a target, opposite, left and right. During the training trials, the animals were trained to find the hidden platform in the pool for four consecutive days, four trials per day. The mice were gently placed into the water facing the pool wall at one of four imaginary starting positions (target, opposite, left and right) around the perimeter of the pool. The trials were performed for up to a maximum of 90 s. If the mouse reached the platform within 90 s, it was allowed to remain there for 15 s; if not—mouse was guided to the platform and then allowed to rest on the platform for 15 s. The inter-trial interval was at least 20 min. After every trial, mice were placed in a drying cage and allowed to dry before they were returned to their home cages. The results were expressed as latency in finding the hidden platform. Probe trials were performed 24 h and 6 days after the last training trial with removal of the platform from the pool. All mice began swimming from a position opposite the target quadrant and were allowed to spend 90 s in the pool. In the probe trial, time in the target quadrant and swimming distance were analyzed using EthoVision video tracking system (version XT 11.5, Noldus Information Technology, Wageningen, Netherlands).

#### Tail suspension test

Depression-like behavior was assessed by tail suspension test, as previously described (21). Briefly, each animal was suspended with tape (17 cm) from a horizontal rod elevated 30 cm above a clean cage for 6 min. To prevent mice from climbing their tails, a clear hollow cylinder (Ø = 4.5 cm, H = 5.5 cm) was placed around the tail before the suspension. Mice were recorded for 6 min using the digital HD video camera recorder (Handycam HDR-CX11E, Sony Corporation, Tokyo, Japan), and immobilization was analyzed during the last 4 min.

#### Open-field test

Locomotor activity was assessed by an open-field test. The open-field was a square arena (44 × 44 cm) shielded by 30 cm high opaque walls. The Square area (20 × 20 cm) was defined as the center. The mouse was gently placed in the center of the field and allowed to explore for 12 min. The distance traveled, velocity and time spent in the center were recorded and analyzed for 4 min sessions using the EthoVision video tracking system (version XT 11.5, Noldus Information Technology, Wageningen, Netherlands).

### Statistical analysis

The statistical calculations were performed using the GraphPad Prism 8.1 software (Graph Pad Prism software, Inc., La Jolla, CA, USA). The Shapiro-Wilk test was used to test the distribution of the data. A two-way mixed-design analysis of variance with repeated measures (two-way RM ANOVA) was used to calculate group differences at each time-point, which included one between-subjects variable (latFPI vs. sham) and one within-subjects variable (time). In all comparisons, Fisher Least Significant Difference (LSD) *post-hoc* analysis was used when appropriate (one or both main factors were statistically significant). Area under curve (AUC) values were compared with an ordinary one-way ANOVA. Statistical outliers were identified using Grubbs' test (α = 0.05). Two statistical outliers were removed from the sham group at 3 month-time point in acetone and MWM tests. All data are presented as mean ± standard error means (SEM). *P*-values <0.05 were considered significant.

## Results

### latFPI impairs sensorimotor ability up to 12 months post-injury

**Figure 2:**
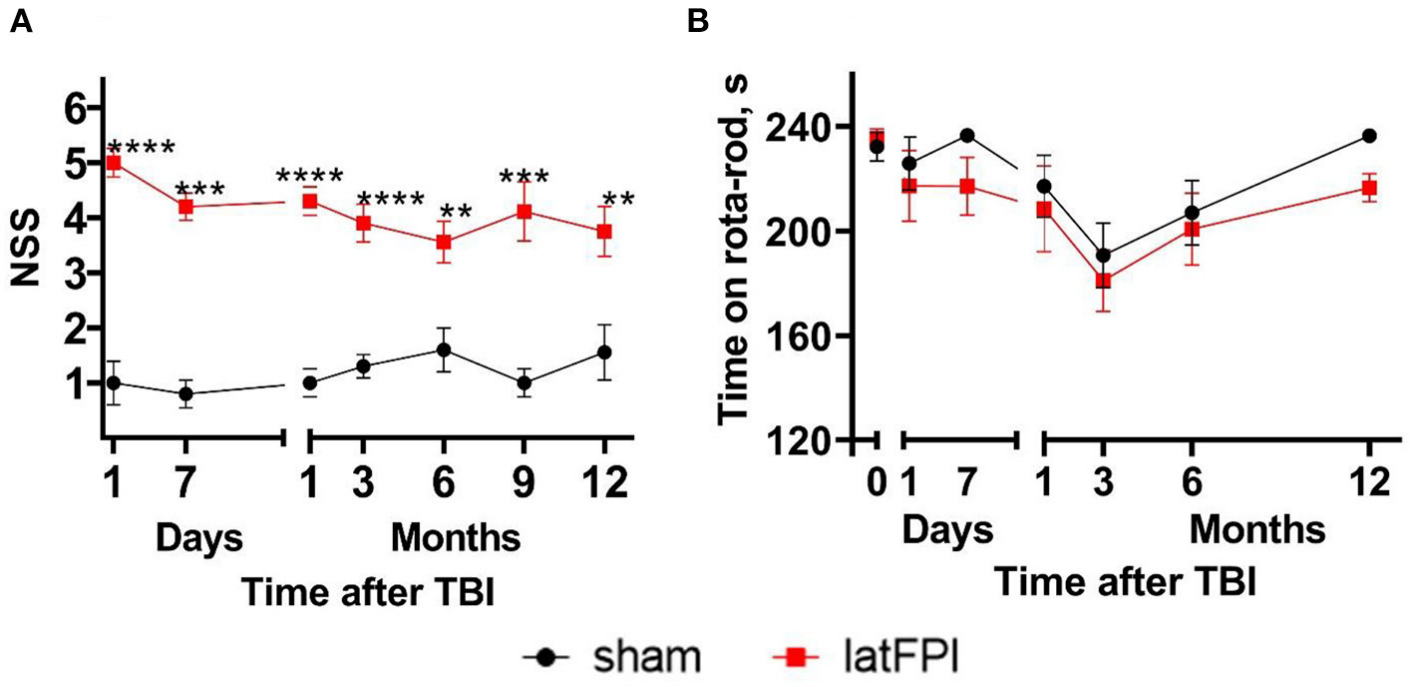
Neurological status of mice following latFPI. Brain-injured animals showed significant neurological impairments up to 12 months post-injury compared to sham-operated animals **(A)**. Motor coordination was not affected by latFPI during the 12-month assessment period **(B)**. Data are expressed as mean ± SEM. **p < 0.01; ***p < 0.001; ****p < 0.0001 vs. sham group (two-way RM ANOVA followed by Fisher's LSD test).

Sensorimotor function was assessed using the NSS. The NSS was significantly higher for the latFPI group than for the sham-operated mice up to 12 months post-injury (*p* < 0.0001, Figure 2A). Brain-injured animals mostly failed in grip strength and balance tasks. The accelerating rotarod test was used to monitor motor coordination throughout the study. Time on rotarod was not significantly different between latFPI and sham animals at any time point post-injury (Figure 2B).

### latFPI induces chronic cold and mechanical sensitivity

**Figure 3:**
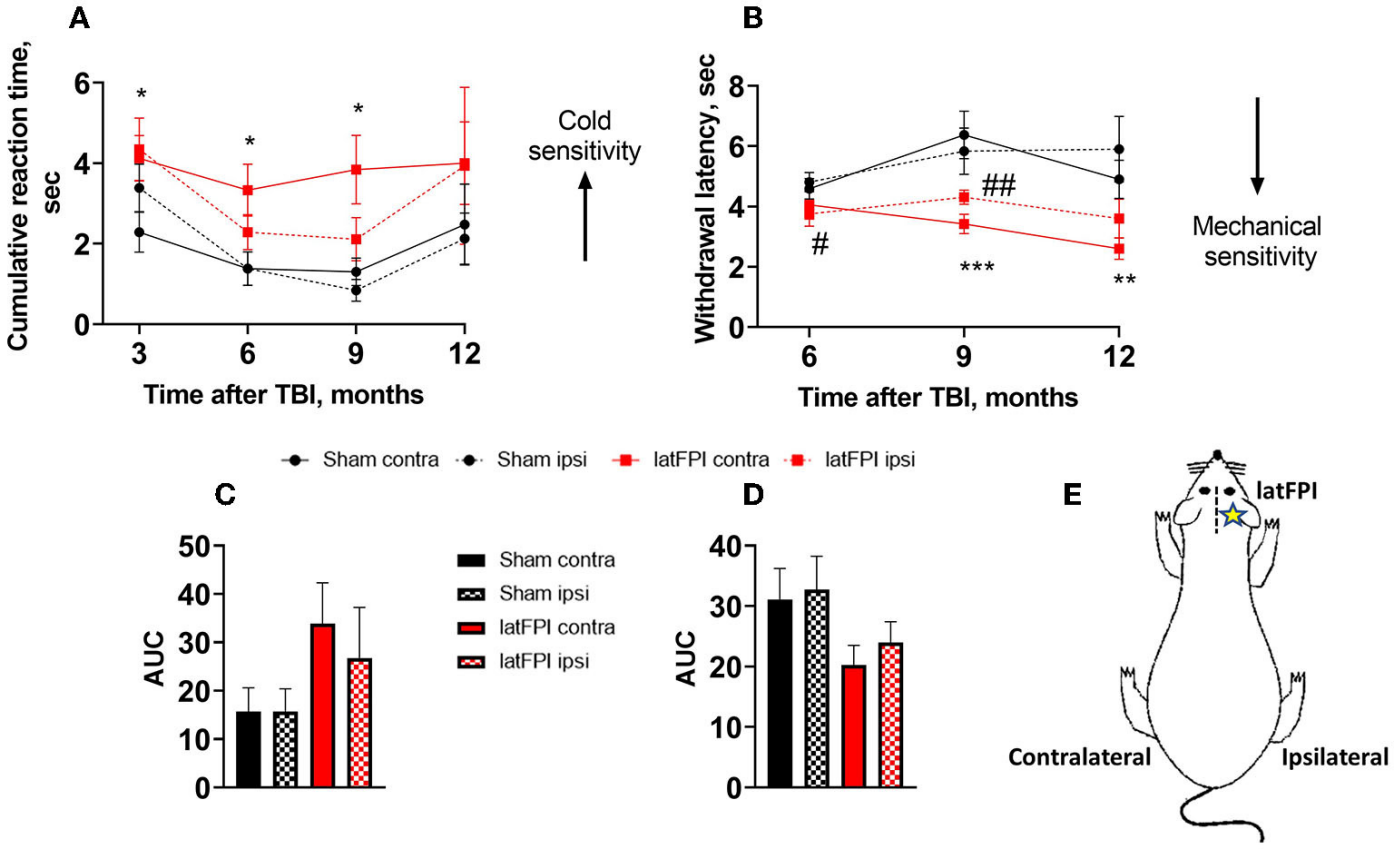
Cold and mechanical sensitivity following latFPI in mice. The cumulative reaction time of contralateral and ipsilateral hind paw licking or shaking in the acetone test **(A)** and latency of contralateral and ipsilateral hind paw withdrawal in the electronic von Frey test **(B)** were recorded 3, 6, 9, and 12 months post-injury. Data are expressed as mean ± SEM. *p < 0.05; **p < 0.001 contralateral latFPI vs. contralateral sham hind paw. #*p* < 0.05; ##*p* < 0.001 ipsilateral latFPI vs. ipsilateral sham hind paw (two-way RM ANOVA followed by Fisher's LSD test). The area under the curve (AUC) in the acetone **(C)** and von Frey tests **(D)**. The AUC was calculated over the whole post-injury period. Schematic drawing of contralateral and ipsilateral sides. The contralateral hind paw corresponds to the opposite side of the lesion, and the ipsilateral hind paw corresponds to the lesion side **(E)**. Data are expressed as mean ± SEM. *p < 0.05; **p < 0.01; ***p < 0.01 vs. sham group (one-way ANOVA followed by Dunnett's test).

Peripheral cold sensitivity was evaluated by acetone test. The cumulative reaction time of the contralateral hind paw (opposite side to the lesion) was increased in the latFPI group compared to the sham group at 3 months (4.1 ± 0.6 s vs. 2.3 ± 0.5 s, *p* = 0.026), 6 months (3.3 ± 0.7 s vs. 1.0 ± 0.2 s, *p* = 0.007) and 9 months post-injury (3.8 ± 0.9 s vs. 1.2 ± 0.2 s, *p* = 0.014, Figure 3A). The AUC was not significantly different between latFPI (34 ± 9 for contralateral, 27 ± 10 for ipsilateral hind paw) and sham mice (16 ± 5 for contralateral, 16 ± 5 for ipsilateral hind paw, *p* = 0.234, Figure 3C).

Peripheral mechanical sensitivity was evaluated by the von Frey test. A significant decrease in the mechanical withdrawal latency of the contralateral hind paw was observed in the latFPI group compared to the sham group at 9 months (3.4 ± 0.3 s vs. 5.9 ± 0.7 s, *p* = 0.001) and 12 months post-injury (2.6 ± 0.4 s vs. 4.9 ± 0.4 s, *p* = 0.0113, Figure 3B). The withdrawal latency was also significantly decreased in the ipsilateral hind paw in latFPI mice compared to sham mice 12 months post-injury (3.6 ± 0.6 s vs. 5.9 ± 0.7 s, *p* = 0.0157, Figure 3B). The AUC was not significantly different between latFPI (20 ± 3 for contralateral, 24 ± 4 for ipsilateral hind paw) and sham animals (31 ± 5 for contralateral, 33 ± 5 for ipsilateral hind paw, *p* = 0.1627, Figure 3D).

### latFPI results in progressive depression-like behavior

**Figure 4:**
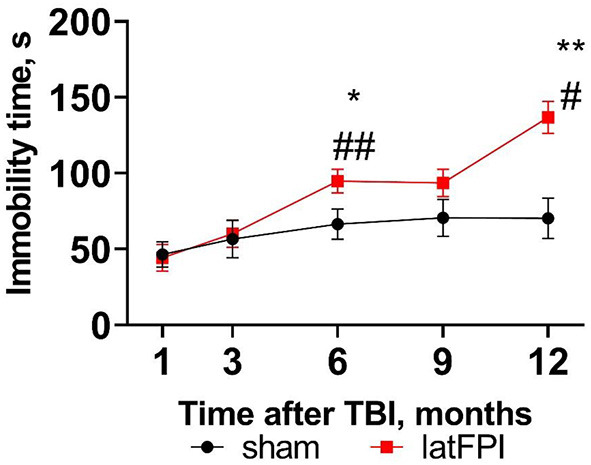
Depressive-like behavior was determined by the time spent immobile in the tail suspension test 1, 3, 6, 9, and 12 months after latFPI. Data are expressed as mean ± SEM. *p < 0.05, **p < 0.01 vs. sham, #*p* < 0.05, ##*p* < 0.001 compared to 1 month (two-way RM ANOVA followed by Fisher's LSD test).

Depressive-like behavior was assessed by the tail suspension test. Immobility time was significantly increased in the latFPI group 6 months (94.7 ± 7.9 vs. 66.4 ± 10.0, *p* = 0.040) and 12 months (136.8 ± 10.5 vs. 70.2 ± 13.3, *p* = 0.001, *p* = 0.001) post-injury compared to the sham group (Figure 4). Throughout the experiment, brain-injured animals displayed significantly longer immobility times starting at 6 months compared to 1 month post-injury (*p* = 0.009, Figure 4). Immobility time remained unchanged over time in sham animals (Figure 4).

### latFPI produces no spatial learning and memory impairments

**Figure 5:**
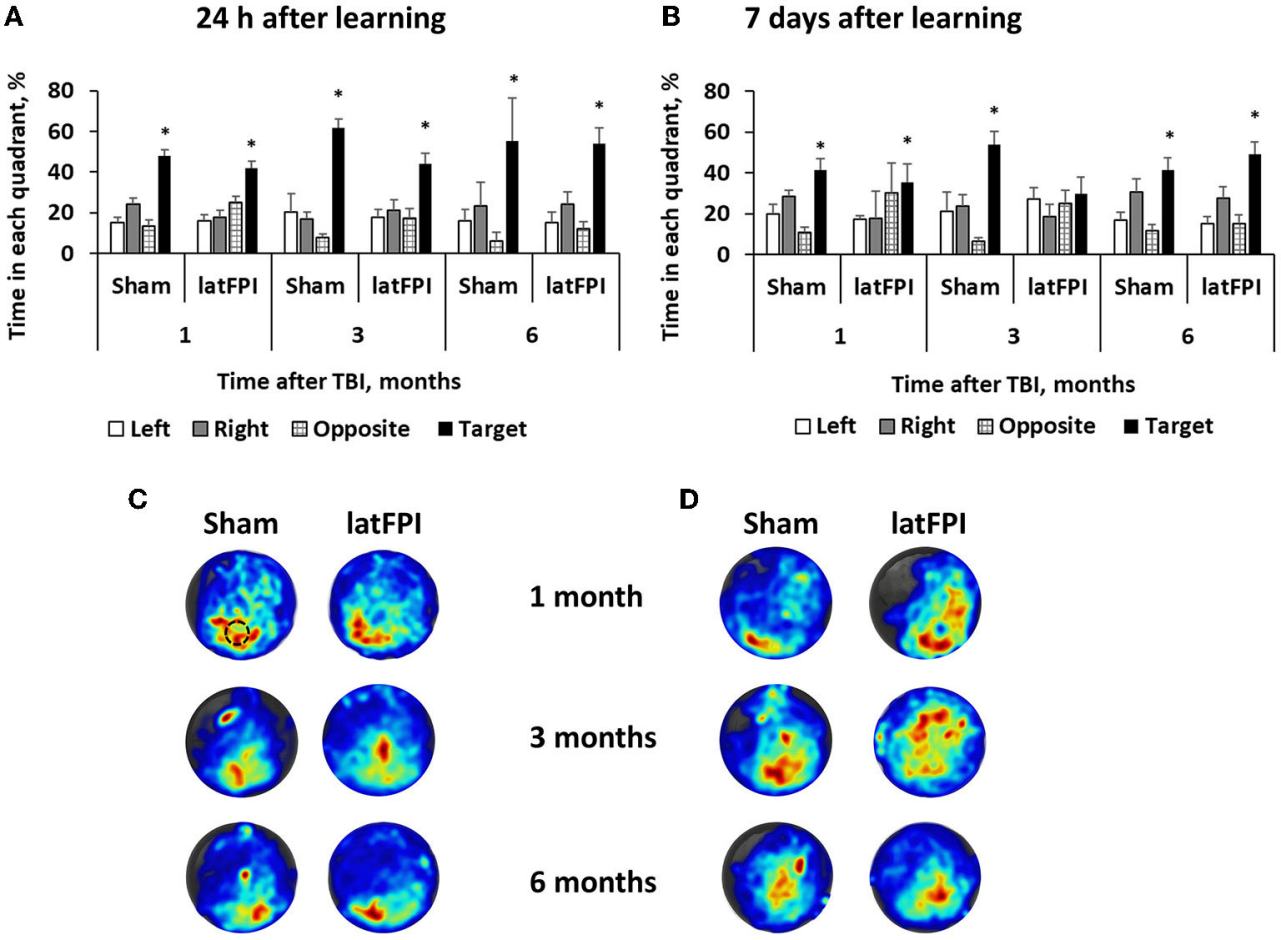
Assessment of spatial memory by the Morris water maze 1, 3 and 6 months after latFPI. Percentage of time spent in each quadrant 24 h **(A)** and 7 days **(B)** after the last training day. Heatmaps representing weighted occupancy across the entire 90 s probe trial 24 h **(C)** and 7 days **(D)** after the last training day 1, 3, and 6 months post-injury. Hot colors indicate longer dwell times. The platform area is denoted with a black circle. Data are expressed as mean ± SEM for the entire 90 sec, *p < 0.05 target quadrant vs. at least 2 other quadrants (one-way ANOVA followed by Dunnett's multiple comparison test).

MWM was performed to evaluate spatial learning and memory. During the learning trials, no differences in latency to reach the platform were seen between groups, indicating comparable learning of the task (data not shown). During the probe trial, which was carried out 24 h following the last day of learning, both groups spent a similar amount of time in the target quadrant (Figure 5A). In addition, both groups showed a preference for the target quadrant compared to other quadrants of the pool (Supplementary Figure S1A), indicating that latFPI exposure did not impair hippocampal-dependent memory of a previously learned platform location at any time point. At 7 days following the last day of learning, brain-injured mice did not show a preference for any of the quadrants 3 months after injury (*p* > 0.05 for target vs. other quadrants, Figure 5B). There was a trend toward decreased time spent in the target quadrant in latFPI animals (*p* = 0.0565); however, the difference failed to reach significance between groups (mean % 54 ± 6 for sham vs. 30 ± 8 for latFPI, Figure 5B). No differences in any evaluated parameters were seen at 1- and 6-months post-injury. There were no differences between groups in swimming distance, suggesting no effect on enhanced or impaired physical function that could account for group differences (Supplementary Table S2).

**Figure 6:**
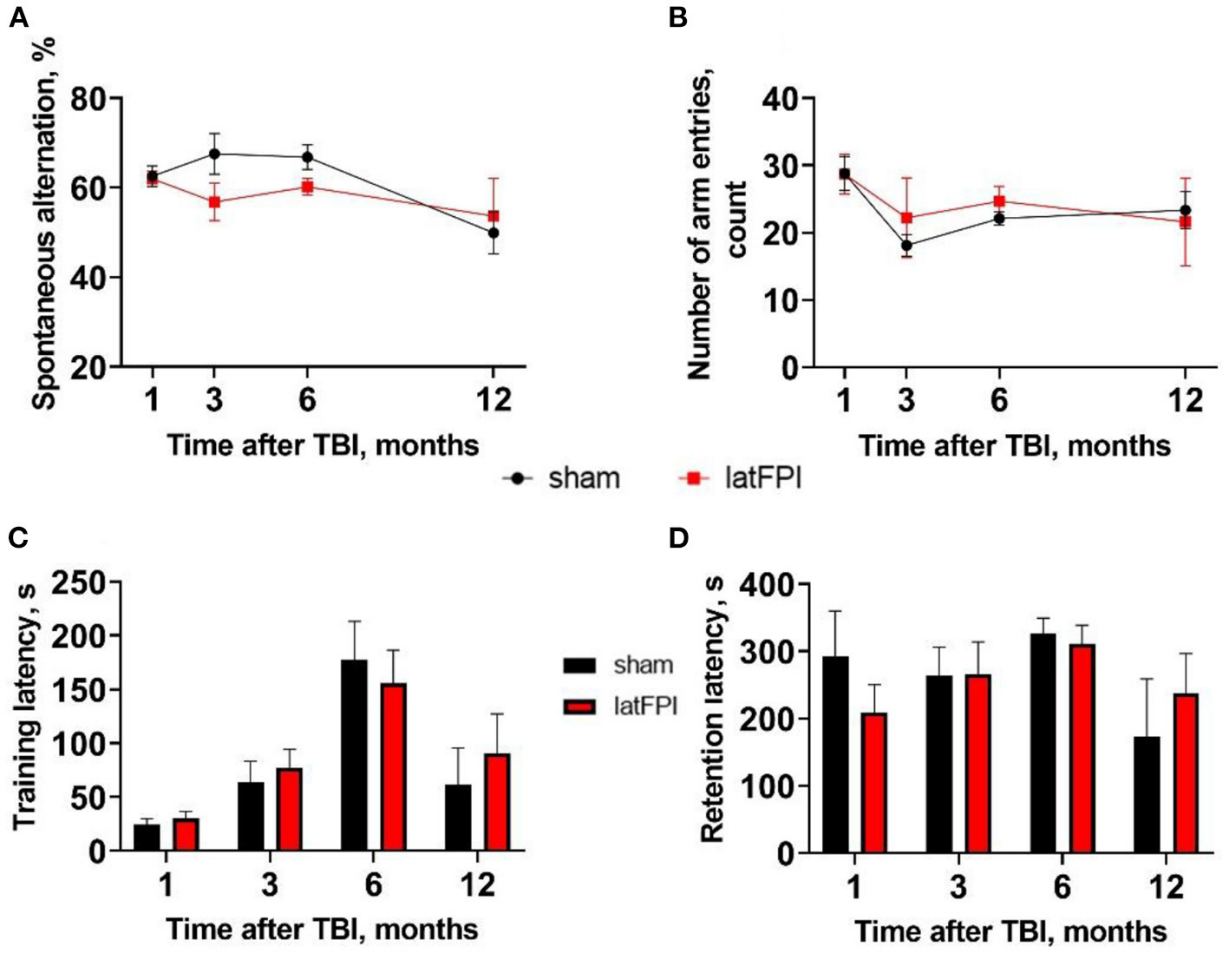
Assessment of working and contextual memory after latFPI. Spontaneous alternation behavior **(A)** and the number of arm entries **(B)** in the Y-maze test 1, 3, 6, and 12 months after latFPI. Latency time to enter dark compartment on the training **(C)** and retention day **(D)** in the passive-avoidance test 1, 3, 6, 12 months after latFPI in mice. Data are expressed as mean ± SEM (two-way RM ANOVA followed by Fisher's LSD test).

Y-maze testing revealed no significant changes in spontaneous alterations or total arm entries, indicating no impairments in working memory (Figures 6A,B). Likewise, there was no significant effect of injury on contextual memory assessed by the passive avoidance test. Sham and TBI mice exhibited a similar time to enter the dark compartment 24 h after learning up to 12 months post-injury (Figure 6D). We observed increased step-through latency on the learning day over time, suggesting potential habituation due to repeated measures in the same environment (Figure 6C).

### latFPI has no impact on general health and activity

No animal subjected to sham injury was excluded. The length of the apnea for the animals subjected to latFPI was 47.2 ± 6.7 s. The body weight was slightly decreased up to 3 days post-injury for latFPI mice compared to sham mice, but there were no differences between groups during the whole period (Supplementary Figure S1A). Some mice were lost throughout the year of the study, but the deaths were spontaneous (one from the sham group and three from the TBI group) (Supplementary Figure S1B). latFPI mice showed no locomotion deficits within 12 months post-injury. In the open field test, brain-injured mice were more active at 6 months post-injury than sham mice (*p* = 0.004), although this effect had dissipated at 9 and 12 months post-injury (Supplementary Table S3). In the Y-maze test, the number of arm entries in the latFPI group was similar to that in the sham group (Supplementary Table S2, Figure 6B).

## Discussion

**Figure 7:**
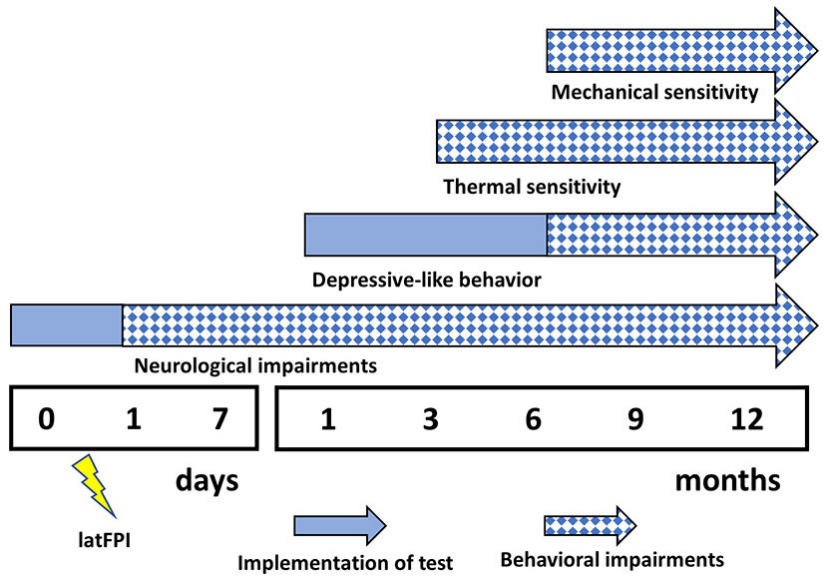
Summary of behavioral impairments following latFPI. Neurological impairments were evaluated by neurological severity score, depressive-like behavior by tail suspension test, thermal and mechanical pain sensitivity by acetone and von Frey test, respectively.

In the present study, we demonstrated that brain injury to mice results in long-lasting and continually evolving alterations in behavior (Figure 7). Over time, a single latFPI induced persistent cold and mechanical peripheral pain up to 12 months after injury. Our experimental data also revealed that TBI increased immobility time in the tail suspension test, suggesting the development of depression-like behavior. This is the first study to examine the prevalence of persistent peripheral pain over long-term follow-up in mice after experimental TBI, suggesting that latFPI in mice provides a suitable platform to investigate the biological mechanisms of peripheral pain and depression following TBI.

Among the long-term consequences of TBI, one of the most recently debated yet severely understudied is pain (7). Pain is an acute response to brain injury and typically lasts several weeks in patients (8). However, in a small group of patients, pain persists beyond the healing of damaged tissue and becomes chronic (7). The most common long-term pain condition reported in TBI patients is a posttraumatic headache, and in most cases, it is associated with a direct brain tissue injury (5, 7). However, headache is not the only type of pain present after TBI. Clinical studies indicate that chronic pain is located in body regions that have not been injured during trauma, such as the back and lower extremities (8, 35, 36). The mechanisms that drive the development of chronic pain are not well-understood, whether the pain is due to neuropathy, central pain, or secondary to direct tissue injury. Despite emerging evidence that non-head pain is common after TBI, only recently have animal studies begun to explore the mechanisms supporting the development of pain after TBI. Moreover, these studies were limited by short-term follow-up assessment points and pain evaluation in periorbital regions (12, 37–41). A few studies have examined pain-like behavior in peripheral regions, such as hind paws (39, 42, 43). Increased mechanical sensitivity has been observed within 3 weeks after TBI, suggesting that these animals experience acute pain associated with disrupted communication between the brain and spinal cord (43). Here, we showed that latFPI causes cold and mechanical sensitization in body regions distant from the central nervous system. Namely, mechanical sensitivity was observed in contralateral and ipsilateral hind paws starting at 6 months after TBI. Interestingly, cold sensitivity was observed only in the contralateral hind paw, and significant differences were observed starting 3 months after injury. Clinical research shows that sensation to thermal and tactile stimulation tends to be unilateral, mainly located on the side of the body that was contralateral to the TBI side (8). There is evidence that the surgical procedure per se may lead to increased pain sensitivity in the periorbital and plantar regions in rats (12) and mice (38). However, these findings have been observed at early time points post-TBI and could be explained by direct tissue damage and the inflammatory response to injury.

It is noteworthy that both pain and depression are often cooccurring after TBI, and a significant predictor of persistent pain in patients is an early presence of depressive symptoms after TBI (44). Depressive-like symptoms following TBI have commonly been reported as comorbidities of posttraumatic pain (5, 45). In our study, moderate latFPI induced progressive depressive-like behavior starting at 6 months post-injury. Since the latFPI and sham groups did not differ in locomotor activity in the open field, Y-maze or MWM, the increased immobility time in the tail suspension test reflected the increase in depressive-like behavior. These data are consistent with recently published results in mice suffering from progressive and severe depression-like behavior 6 months after CCI (16). Our previous findings revealed that moderate latFPI also induces depressive-like behavior in male CD-1 mice (21). However, there are also reports where depression-like behavior is not observed within 12 months after closed head injury (46). Our results suggest that moderate latFPI induces progressive depression-like behavior, and this model is suitable for studying common clinical symptoms of depression that are diagnosed following TBI.

Assessment of learning and memory is widely used in preclinical research to determine the duration and severity of cognitive impairments following TBI. Cognitive deficits in experimental TBI models have been observed up to 1 year or longer after the initial injury (Supplementary Table S1). In these long-term studies, cognitive impairments have been observed after severe TBI accompanied by extensive brain tissue loss (13, 15, 16, 47, 48). In cases where brain tissue damage is not extensive (e.g., tissue loss in the ipsilateral cortex), cognitive impairments are less prominent or not observed (17, 21). In the present study, we did not observe cognitive impairments in any of the evaluated memory tests. Hippocampal-dependent cognitive tasks, such as spatial learning and memory, were not affected up to 6 months post-injury. Only one study showed that hippocampal-dependent learning tasks were affected from 2 to 12 months after latFPI in rats; however, the injury was accompanied by progressive tissue loss resembling severe brain injury (18). While some speculate that water maze performance and sensitivity depend on the difficulty of the protocol (49), we believe that the moderate latFPI model is not suitable to study memory deficits after TBI in mice. Furthermore, we were unable to find any differences between the performance of injured and sham mice on the Y-maze and passive avoidance tests, suggesting that moderate latFPI in the present study did not produce cognitive impairments. It is important to note that localization of craniotomy is crucial while performing latFPI. Even though the surgery is performed similarly between animals, medial and rostral shifts can worsen or lessen injury-dependent hippocampal damage (50).

Of note, using only male mice in our study may be considered as a limitation. The incidence of TBI is higher in male than females, however, the consequences of those injuries may be different for the sexes in both preclinical and clinical studies (51). In particular, it has been reported that women have higher risk for persistent pain and depression after TBI (44, 52, 53). There are concerns that estrous cycle changes may introduce variability in the development of neurological impairments, although this has been debated. Previous preclinical study has failed to identify sex-linked differences in nociceptive sensitization and depressive-like behavior after closed head injury (46, 54). However, this should not be taken as underestimating the importance of sex differences in preclinical TBI research and further studies are needed.

The chronic peripheral pain and depression observed in the present study underlie the clinical importance of severe and long-lasting consequences after TBI. Nevertheless, pain in body regions other than the head is often not assessed systematically in clinical and preclinical TBI research. It is essential that this factor is taken into consideration in evaluating posttraumatic pain. Patients with TBI may benefit from timely assessment and intervention to minimize the development and impact of pain. Acute and continued pain management may be paramount for addressing depression or other neurological impairments in TBI patients.

## Data availability statement

The raw data supporting the conclusions of this article will be made available by the authors, without undue reservation.

## Ethics statement

The animal study was reviewed and approved by Latvian Animal Protection Ethical Committee of Food and Veterinary Service in Riga, Latvia.

## Author contributions

Conceptualization: LZ and MD. Methodology, validation, formal analysis, and data analysis and interpretation: GS, BS, EV, and LZ. Behavioral analysis: GS and BS. Writing—original draft preparation: GS and LZ. Writing—review and editing: GS, BS, EV, ID, LZ, and MD. All authors have read and agreed to the published version of the manuscript.

## Funding

This study was supported by the framework of EU-ERA-NET NEURON CnsAflame and TRAINS.

## Conflict of interest

The authors declare that the research was conducted in the absence of any commercial or financial relationships that could be construed as a potential conflict of interest.

## Publisher's note

All claims expressed in this article are solely those of the authors and do not necessarily represent those of their affiliated organizations, or those of the publisher, the editors and the reviewers. Any product that may be evaluated in this article, or claim that may be made by its manufacturer, is not guaranteed or endorsed by the publisher.
