# Supplementary material for: Moderate traumatic brain injury triggers long-term risks for the development of peripheral pain sensitivity and depressive-like behavior in mice
Source: Front Neurol. 2022 Sep 20;13:985895. doi: 10.3389/fneur.2022.985895 (PMC9531915; doi:10.3389/fneur.2022.985895)
Supplement: Supplementary file 1 [file Data_Sheet_1.docx]

Supplementary Material

**
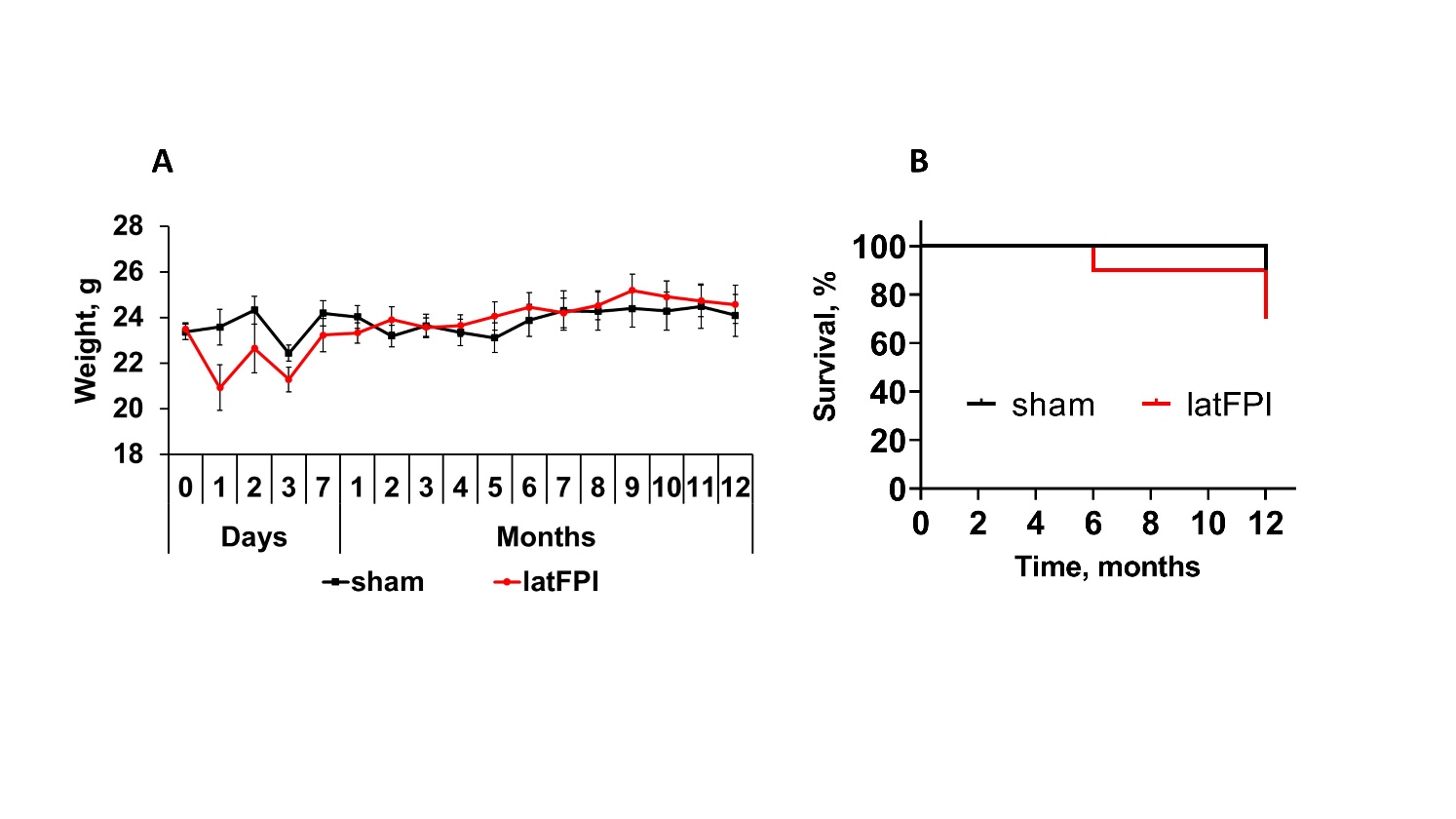
**

**Supplementary Figure 1.** General health monitoring after TBI. Relative body weight changes (A) and mortality (B) after latFPI. Data are expressed as mean ± SEM (two-way RM ANOVA followed by Fisher's LSD test). The difference in overall mortality was calculated using the chi-square test.

**Supplementary Table 1.** Overview of the follow-up studies characterizing long-term neurobehavioral changes at or beyond 6 months after CCI and FPI brain injury models.

| Strain | Time after TBI | Sensorimotor deficits | Depressive-like behavior | Anxiety-like behavior | Cognitive impairments | Brain structural changes | Reference |
| --- | --- | --- | --- | --- | --- | --- | --- |
| Mice, CCI | | | | | | | |
| C57BL/6 | 1 week – 12 MPI | Yes, up to 12 MPI  (NSS, BW) | - | - | Yes, up to 6 MPI (MWM) | Tissue loss in il cortical, hippocampal, thalamic and striatum regions | (Pischiutta et al., 2018a) |
| C57BL/6 | 1 day – 12 MPI | Yes, up to 12 MPI  (BW) | Yes, up to 12 MPI (TST) | - | Yes, up to 12 MPI (BM) | Tissue loss in il cortical, hippocampal, thalamic and striatum regions | (Mao et al., 2020) |
| C57BL/6N | 1 day –  225 days | Yes, at 21 DPI (RR), up to 191 DPI (catwalk) and 225 DPI (NSS) | - | Yes, at 184 DPI (EPM) | Yes, up to 205 DPI (MWM)  No (FC) | Tissue loss in il cortical and hippocampal regions | (Pöttker et al., 2017) |
| C57BL/6N | 2 weeks – 20 MPI | - | - | - | Yes, at 20 MPI (FC) | Tissue loss in il cortical, hippocampal, thalamic and striatum regions | (Campos-Pires et al., 2019) |
| C57BL/6J | 1 week – 12 MPI | Yes, up to 12 MPI (RR) | - | - | Yes, up to 12 MPI (MWM) | Tissue loss in il cortical, hippocampal and thalamic regions | (Shear et al., 2004) |
| Swiss | 15 days -10 MPI | Yes, up to 9MPI (pole test) | - | No (EPM) | No (BM) | Tissue loss in il cortical region | (Leconte et al., 2020) |
| Mice, LFPI | | | | | | | |
| CD-1 | 1 day – 12 MPI | Yes, up to 12 MPI (NSS). No (RR) | No (TST) | No (EPM) | Yes, at 7 MPI (BM)  No (y-maze) | - | Stelfa et al., 2021 |
| Rats, CCI | | | | | | | |
| Sprague–Dawley | 3-6 MPI | Yes, up to 6MPI (RR)  Yes, up to 5MPI (FF) | - | No (ZM) | Yes, up to 5 MPI (MWM) | Tissue loss in the cortical region. Disrupted corpus callosum | (Kamper et al., 2013) |
| Sprague-Dawley | 2 weeks – 12 MPI | - | - | - | Yes, at 12 MPI (MWM) | Tissue loss in il cortical region. | Dixon et al., 1999 |
| Rats, LFPI | | | | | | | |
| Sprague-Dawley | 24 h – 12 MPI | Yes, up to 2 MPI (NSS) | - | - | Yes, up to 12 MPI (learning trial MWM). No (probe trial MWM) | Tissue loss in il cortical region. | Pierce et al., 1998 |

MPI-months postinjury, CCI-cortical impact injury, LFPI-lateral fluid percussion injury, NSS-neurological severity score, RR-rotarod, MWM-Morris water maze, TST-tail suspension test, BW-beam walk, BM-Barnes maze, EPM-elevated plus maze, FC-fear conditioning, ZM-zero maze, OF-open field, FF-foot fault, AT-actimetry test, il-ipsilateral, “-”not performed.

**Supplementary Table 2.** The total swimming distance (cm) in the MWM test 1, 3 and 6 months after latFPI (two-way RM ANOVA followed by Fisher's LSD test).

| Months | 1 | | 3 | | 6 | |
| --- | --- | --- | --- | --- | --- | --- |
| Probe | 24 h | 6 days | 24 h | 6 days | 24 h | 6 days |
| sham | 1265 ± 156 | 1265 ± 156 | 1144 ± 161 | 1234 ± 169 | 1113 ± 103 | 1389 ± 116 |
| latFPI | 1513 ± 101 | 1512 ± 106 | 1295 ± 139 | 1249 ± 140 | 1249 ± 136 | 1296 ± 136 |

**Supplementary Table 3.** The total moved distance (cm) in an open-field test 6, 9 and 12 months after latFPI. Data are expressed as mean ± SEM. ** *p* < 0.01 vs. sham group (two-way RM ANOVA followed by Fisher's LSD test).

| Months | 6 | 9 | 12 |
| --- | --- | --- | --- |
| sham | 2496 ± 215 | 1783 ± 169 | 2576 ± 398 |
| latFPI | 2984** ± 226 | 2499 ± 174 | 2919 ± 217 |
